# Role of silymarin as antioxidant in clinical management of chronic liver diseases: a narrative review

**DOI:** 10.1080/07853890.2022.2069854

**Published:** 2022-05-28

**Authors:** Alessio Aghemo, Olga P. Alekseeva, Francesco Angelico, Igor G. Bakulin, Natalia V. Bakulina, Dmitry Bordin, Alexey O. Bueverov, Oxana M. Drapkina, Anton Gillessen, Elvira M. Kagarmanova, Natalia V. Korochanskaya, U. A. Kucheryavii, Leonid B. Lazebnik, Maria A. Livzan, Igor V. Maev, Anatolii I. Martynov, Marina F. Osipenko, Evgenii I. Sas, Antonina Starodubova, Yurii P. Uspensky, Elena V. Vinnitskaya, Emilia P. Yakovenko, Alexey A. Yakovlev

**Affiliations:** a Department of Biomedical Sciences, Humanitas Research Hospital IRCCS, Rozzano, Italy; b Gastroenterological Center, Semashko National Research University, Moscow, Russia; c Sapienza School of Medicine, Rome, Italy; d Department of Propaedeutics of Internal Diseases, Federal State Medical University of Ministry of Health of Russia, Chief Specialist-Therapist of the North-Western Federal district, Moscow, Russia; e Department of Therapy and Clinical Pharmacology, North-Western State Medical University, Moscow, Russia; f Department of Pancreatic, Biliary, and Upper Digestive Tract Disorders, A.S. Loginov Moscow Clinical Scientific Center, Moscow, Russia; g Department of Gastroenterology and Hepatology, Moscow Medical Academy, Moscow, Russia; h Ministry of Health of the Russian Federation, Chief Specialist of Therapy and General Practice Ministry of Health of Russia, Grozny, Russia;; i Department of Internal Medicine, Herz-Jesu-Hospital, Muenster, Germany; j Gastroenterological Department, GBUZ RB City clinical Hospital, Sterlitamak, Russia; k Gastroenterology Center, Regional Clinical Hospital, Tyumen, Russia; l Department of Propaedeutics of Internal Diseases and Gastroenterology, Moscow State University of Medicine and Dentistry, Moscow, Russia; m Department of Polyclinic Therapy, Moscow State University of Medicine and Dentistry, Moscow, Russia; n Department of Faculty Therapy, Omsk State Medical University, Omsk, Russia; o Department of Propedeutics of Internal Diseases and Gastroenterology, Moscow State University of Medicine and Dentistry, Moscow, Russia; p Department of Internal Diseases, Moscow State University of Medicine and Dentistry, Moscow, Russia; q Department for Science, Innovations and Informatization, Novosibirsk State Medical University, Novosibirsk, Russia; r 2nd Department of Therapy, Ministry of Defense of the Russian Federation, Moscow, Russia; s Department of Scientific and Clinical Work, INSTITUTE "Federal Research Center of Nutrition and Biotechnologies", Moscow, Russia; t Department of faculty therapy, Saint Petersburg State Pediatric Medical University (Spbpgmu) of the RF MOH, St. Petersburg, Russia; u Department of Hepatology, Moscow Clinical Research and Practice Center, Moscow, Russia; v Department of Gastroenterology, Faculty of Advanced Medical Education of the Russian National Research Medical University, Moscow, Russia; w Department of gastroenterology and endoscopy, Rostov State Medical, Rostov, Russia

**Keywords:** Chronic liver disease, non-alcoholic fatty liver disease, alcoholic fatty liver disease, metabolic-associated fatty liver disease, drug-induced liver injury, oxidative stress, silymarin, hepatoprotective and hepatotropic effects

## Abstract

Chronic liver disease (CLD), manifested as hepatic injury, is a major cause of global morbidity and mortality. CLD progresses to fibrosis, cirrhosis, and—ultimately—hepatocellular carcinoma (HCC) if left untreated. The different phenotypes of CLD based on their respective clinical features and causative agents include alcoholic liver disease (ALD), non-alcoholic fatty liver disease (NAFLD), metabolic-associated fatty liver disease (MAFLD), and drug-induced liver injury (DILI). The preferred treatment modality for CLD includes lifestyle modification and diet, along with limited pharmacological agents for symptomatic treatment. Moreover, oxidative stress (OS) is an important pathological mechanism underlying all CLD phenotypes; hence, the use of antioxidants to manage the disease is justified. Based on available clinical evidence, silymarin can be utilized as a hepatoprotective agent, given its potent antioxidant, antifibrotic, and anti-inflammatory properties. The role of silymarin in suppressing OS has been well established, and therefore silymarin is recommended for use in ALD and NAFLD in the guidelines approved by the Russian Medical Scientific Society of Therapists and the Gastroenterology Scientific Society of Russia. However, to discuss the positioning of the original silymarin in clinical guidelines and treatment protocols as a hepatoprotective agent for managing CLD concomitantly with other therapies, an expert panel of international and Russian medical professionals was convened on 11 November 2020. The panel reviewed approaches for the prevention and treatment of OS, existing guidelines for patient management for CLD, and available evidence on the effectiveness of silymarin in reducing OS, fibrosis, and hepatic inflammation and presented in the form of a narrative review.

**Key messages**
- An expert panel of international and Russian medical professionals reviewed existing guidelines for ALD, NAFLD, MAFLD, and DILI to establish consensus recommendations that oxidative stress is the common pathophysiological mechanism underlying these conditions.
- The panel also discussed the positioning of original silymarin in clinical guidelines and treatment protocols as a hepatoprotective agent for managing CLD concomitantly with other therapies.
- The panel reviewed the effectiveness of 140 mg original silymarin three times a day in reducing oxidative stress in chronic liver diseases such as ALD, NAFLD, MAFLD, and DILI.

## Introduction

Chronic liver diseases (CLDs) are a major cause of global morbidity and mortality, including in the Russian Federation (RF) [1–4]. Several pathophysiological conditions, such as viral infections, alcoholism, genetic inheritance, metabolic abnormalities, autoimmune responses, biliary and vascular conditions, drugs, toxins, environmental pollutants, or even cryptogenic reasons, can lead to hepatic injury. If the injury persists for more than 6 months, it can be classified as CLD, which may eventually progress to cirrhosis or hepatocellular carcinoma (HCC). Progressive steatosis/steatohepatitis ultimately leads to fibrosis/cirrhosis, which can be caused by exogenous and endogenous factors, such as alcohol, which causes alcoholic liver disease (ALD) or non-alcoholic fatty liver disease (NAFLD) if alcohol was not involved. Steatosis/steatohepatitis can also be due to metabolic dysfunction or medication-induced, which are identified as metabolic-associated fatty liver disease (MAFLD) and drug-induced liver injury (DILI), respectively. In 90% of patients with NAFLD, at least one component of metabolic syndrome is evident, whereas up to 30% of patients with NAFLD show all the components of metabolic syndrome causing MAFLD [5–7].

The diagnosis, clinical phenotype, and treatments for each of these conditions have been elaborated in Russia, the United States, European Union, and Asia Pacific-specific guidelines [5,8–13]. Comorbidities associated with CLD and related hospitalizations have increased over the last decade [1]. In 2017 and 2018, liver fibrosis and cirrhosis were responsible for up to 59% and 55% of all gastrointestinal-related deaths, respectively, in the Northwestern Federal District of Russia. In St. Petersburg, liver diseases caused up to 47% of all gastrointestinal tract-related mortality in 2018 [14]. The presence of CLDs puts a financial and resource burden on patients and health care professionals [15]. Therefore, targeting common pathophysiological mechanisms can be a promising strategy for hepatoprotection, aiming to reduce the clinical and economic burden of CLDs.

Oxidative stress (OS) is the pathological link common to NAFLD, ALD, DILI, and fibrosis [16–18], and it is involved in the initiation and progression of liver diseases. It is primarily attributable to the generation of reactive oxygen species (ROS) and the decrease in endogenous antioxidant defenses [19]. Even though lifestyle interventions such as weight loss, dietary restrictions, and enhanced physical activity are highly recommended, targeting OS during early disease stages could be a useful strategy [20].

Silymarin, also known as *Silybi mariani fructus extractum*, is an effective antioxidant. Its antifibrotic, anti-inflammatory, and hepatoprotective properties have been summarized earlier [21,22]. It was also reported to be safe and well tolerated clinically. However, the level of clinical evidence for efficacy needs to be established, as required by good clinical practice guidelines, primarily because of a lack of standard silymarin across studies and inconsistencies in the dosage or duration of treatment. Nonetheless, positive results about hepatoprotection are available.

Hence, the objective of the present work is to understand the salient features of different phenotypes of CLD and establish the role of OS as a common pathophysiological mechanism involved in CLD. Additionally, the position of original silymarin in the management of CLD has been evaluated, followed by the development of an expert opinion statement on the use of silymarin in CLD.

## Methodology

The review is an outcome of an advisory board meeting involving an expert panel of leading Russian and international gastroenterologists and hepatologists that was convened on 11 November 2020. The panel discussed and evaluated current evidence related to the clinical benefits of silymarin in NAFLD, ALD, and DILI and reviewed the evidence on the effectiveness of silymarin in reducing OS, fibrosis, and inflammation. Additionally, the experts evaluated the existing Russian guidelines for managing NAFLD, ALD, DILI, and liver fibrosis, followed by arriving at a consensus regarding recommendations for the use of original silymarin concomitantly with other therapies. An in-depth literature search was undertaken by the panellists using various databases, such as MEDLINE, PubMed, and Google Scholar, and keywords like “ALD,” “NAFLD,” “MAFLD,” “DILI,” “silymarin,” “CLD,” “Russian guidelines,” and “liver diseases” were used. The references thus obtained were scrutinized by the panellists, and those found relevant, upon evaluation based on the theme of the manuscript, were used for drafting this narrative review.

## Phenotypes of CLDs in Russia

### Alcoholic liver disease

The pathogenesis of ALD includes damage to the mitochondrial membrane along with an increase in lipid peroxidation. This further disrupts the mitochondrial electron transport chain and impairs nicotinamide adenine dinucleotide phosphate hydrogen generation, causing hepatic inflammatory reactions and liver fibrosis [8]. Cessation of alcohol consumption can reverse steatosis. However, chronic steatosis often leads to fibrosis [23].

### Non-alcoholic fatty liver disease

The principal mechanism underlying NAFLD is the accumulation of fat and ROS in liver cells, along with lipid peroxidation [5] in the absence of any secondary cause of hepatic fat [13]. Genetic polymorphisms also play a crucial role in the pathogenesis of NAFLD [5]. Finally, rare causes of NAFLD may be genetic hypobetalipoproteinemia and abetalipoproteinemia [24], as well as lysosomal acid lipase activity deficiency [25]. All individuals affected by NAFLD should also be screened for metabolic syndrome [26]. Lifestyle modification, comprising diet, exercise, and weight loss, is advocated in patients with NAFLD, along with other pharmacological agents [27]. The increasing clinical burden of NAFLD is largely associated with an increased economic burden [7]. Additionally, the association between NAFLD and other liver diseases such as hepatitis C and B viruses has also garnered attention [7].

### Metabolic-Associated fatty liver disease

Of note, MAFLD is a new designation of NAFLD that recognizes metabolic syndrome as an aetiological factor for long-term liver injury. The pathophysiology of MAFLD includes genetic factors, glucotoxicity, and lipotoxicity, where hepatic insulin resistance and inflammation are induced [28]. Major risk factors associated with MAFLD include obesity, type II diabetes mellitus, dyslipidemia, arterial hypertension, metabolic syndrome, insulin resistance, and sedentary lifestyle [11].

### Drug-Induced liver injury

The pathogenesis of DILI includes disruption of oxidation and hydroxylation reactions along with the disruption of the conjugation of drug metabolites with glutathione, sulphate, and glucuronide. The blockage of respiratory chain enzymes also plays a vital role in the development of DILI [9]. Risk factors for DILI principally include age, gender, drug dose, history of drug interactions, obesity, excessive alcohol consumption, diabetes, and chronic kidney disease [9]. The most important step in the management of DILI is the discontinuation of the implicated agent. However, targeted therapies for specific forms of DILI are recommended [29].

### Liver transplant

The presence of NAFLD and NASH is a leading indicator for liver transplantation among adults in the US (21.5%) and Europe (8.4%) [30]. For DILI, close to 10% of cases will eventually need a liver transplant [31,32], while 28.7% of all liver transplants are related to alcohol-associated liver diseases [33]. The rapidly increasing use of liver transplantation for the above indications is associated with pre- and post-transplant management and exerts a pronounced economic, health care, and social burden. Hence, an effective intervention for the prevention and therapy of CLDs is needed. Table 1 presents the different aspects of various phenotypes of CLD.

**Table 1.** The clinical features, aetiology, distinctive features, region specific guidelines, diagnostic and screening criteria, and Russia-specific information on different phenotypes of CLD.

Clinical phenotype	Clinical features	Aetiology	Distinctive features	Diagnostic and screening criteria	Russia-specific information
ALD	The clinical phenotypes of ALD are steatosis (fat deposition), acute and chronic hepatitis (inflammation), and hepatic cirrhosis [8]. Chronic and excessive ethanol consumption leads to ALD, causing damage to the liver parenchyma, which manifests ultimately as cirrhosis. Women and individuals with poor nutrition, genetic polymorphisms or those with hepatotropic viral infections are at a higher risk for developing ALD [8,12,23].	Nearly 60%–100% of people who abuse alcohol develop ALD [8]. An amount of 40–80 g of ethyl alcohol for males and 20 g for females per day has been regarded as hepatotoxic [8].	The morphological spectrum of ALD encompasses macrovesicular or mixed-type steatosis, hepatocellular injury with ballooning, lobular inflammation [12,29].	The most recommended diagnostic techniques for ALD are ultrasound and TE to determine the degree of liver density and associated liver fibrosis [8]. Additionally, liver biopsy confirms the existence of liver injury and alcohol genesis [8]. Furthermore, certain indirect laboratory methods using biological markers for ALD are employed for diagnostic purposes, such as mean corpuscular volume, increased serum AST, increased De Ritis ratio (AST/ ALT ratio), increased serum direct bilirubin, increased serum GGTP, increased CDT [8,12].	According to WHO 2018 data, Russia ranks fourth globally in terms of alcohol consumption. Alcohol is the leading cause of death and disability due to liver cirrhosis and is responsible for 36.7% of liver cirrhosis-related deaths in Russia, which is higher than the global average [14]. Further, some studies have shown that ALD accounts for up to 61% of all hospitalised patients in Russian hepatology departments [8,84]. Nearly 1 in 2 Russians above 18 years of age have alcohol problems and, therefore, are at a high risk of developing alcohol-induced complications. The Russian guidelines do not specify a safe, well-defined daily limit for alcohol consumption.[8]
NAFLD	NAFLD is asymptomatic and comprises a spectrum of clinical conditions involving steatosis, steatohepatitis, fibrosis, and cirrhosis [85]. The histological categorisation of NAFLD includes NAFL and NASH. The presence of ≥5% hepatic steatosis without hepatic ballooning is defined as NAFL, while ≥5% hepatic steatosis with hepatocyte injury and inflammatory with or without fibrosis is NASH [27]. NAFL is a benign condition, while NASH is progressive, often advancing to cirrhosis and HCC [85].	It occurs primarily in individuals who do not consume excessive alcohol [5].	The main morphological criteria for NAFLD include large droplet steatosis, ballooning of hepatocytes, lobular inflammation followed by perisinusoidal fibrosis in later stages [5]. Cardiovascular comorbidities such as atrial fibrillation, ischaemic heart disease, and stroke are common with NAFLD. One-third of patients with NAFLD have a risk of developing liver cirrhosis and HCC. NAFLD also increases the risk of developing diabetes, cardiovascular diseases, chronic kidney diseases, breast and colorectal cancers [5,27,42]. The increase in the global prevalence of diabetes and obesity has also led to a proportionate increase in NAFLD [7].	Ultrasound examination, CT, and MRI are usually utilized for detecting fatty liver [5]. Additionally, non-invasive diagnostic tests such as FibroMax, FibroTest^®^, NAFLD fibrosis score are used to establish the severity of inflammatory changes and stage of liver fibrosis. The existence of steatosis is validated using FLI and NFS [13]. Cytokeratin 18 fragment is used as an inflammatory marker [13]. The elasticity of liver tissue is determined using TE in patients with NAFLD. However, puncture biopsy is considered the gold standard for the diagnosis of NASH [5]. Furthermore, assessment of biochemical parameters—including ALT, AST, GGTP, ALP, total bilirubin, prothrombin, proteinogram, coagulogram—is used to determine the functional state of the liver [5]. Different questionnaires are used to measure patient-related outcomes in NAFLD, including the generic health-related quality-of-life tool, SF-36, and the disease-specific tool the Chronic Liver Disease Questionnaire [7].	The prevalence of NAFLD in Europe is estimated to be about 24% with an increasing gradient from Southern to Northern Europe, and NAFLD is the leading cause of CLD in Russia [5]. According to the 2015 Russian open multicenter prospective screening study, DIREG 2, the prevalence of NAFLD is 37.3% in Russia, depicting an increase of 10% over 7 years, compared to the DIREG 1 report. However, cases of NAFLD-associated liver cirrhosis increased by 5% in Russia during the same period [5].
MAFLD	The diagnosis of MAFLD is based on evidence of hepatic steatosis, in addition to any of 3 criteria—overweight/obesity, type 2 DM, and signs of metabolic dysregulation [86,87].	In individuals with normal body weight with hepatic steatosis, metabolic dysregulation is diagnosed by at least 2 metabolic risk factors (risk factors widely used to identify metabolic syndrome: homeostasis model assessment-estimated insulin resistance score ≥2.5 and plasma hs-CRP level >2 mg/L) [86]. The presence of MAFLD further accelerates the progression of other liver diseases such as ALD [11].	In individuals with normal body weight with hepatic steatosis, metabolic dysregulation is diagnosed by at least 2 metabolic risk factors (risk factors widely used to identify metabolic syndrome: homeostasis model assessment-estimated insulin resistance score ≥2.5 and plasma hs-CRP level >2 mg/L) [86]. The presence of MAFLD further accelerates the progression of other liver diseases such as ALD [11].	A positive diagnosis of MAFLD is based on histological examination (biopsy), imaging, and biomarkers found in fat accumulation in the liver [6]. For the determination of steatosis in MAFLD, ultrasound, FibroScan^®^ vibration-controlled TE-equipped with controlled attenuation parameter, CT, and MRI are used [6]. Certain non-invasive tests such as FLI, AST-to-platelet ratio index, FIB-4, and NFS are also used to determine the existence of steatosis and the severity of fibrosis [11].	–
DILI	Clinical and laboratory variants of DILI include hepatocellular, cholestatic, and mixed [9], depending on the pattern of elevation of liver enzymes [29].	Liver damage attributable to prescription or over-the-counter drugs, including herbal and dietary supplements, is termed DILI [9]. It was commonly associated with anti-TB drugs, but nowadays it is becoming more pronounced with the advent of monoclonal antibody therapies. Antibiotics, cardiovascular drugs, anticancer, nonsteroidal anti-inflammatory agents, and dietary supplements are important drugs more likely to cause DILI [9,33].	The pathogenetic classification of DILI includes direct damage causing intrinsic or idiosyncratic DILI. Intrinsic DILI depends on a specific drug causing dose-dependent hepatotoxicity. It occurs in a large proportion of individuals exposed to the drug (predictable) and within a short time span (hours to days). Idiosyncratic DILI is more frequent and occurs only very rarely among treated patients (unpredictable), and often only after several months of treatment [9].	Causality scores such as the RUCAM are used for confirmation of DILI [9,88]. The diagnosis varies from physical examination, laboratory findings, and instrumental diagnostics [9]. Serum ALT/AST, ALP, and total bilirubin levels form the primary parameters for detecting and classifying liver damage in suspected DILI [29]. Additionally, tests for hepatitis C virus RNA and anti-hepatitis E virus IgM are recommended in patients with suspected DILI, to eliminate the possibility of acute hepatitis C and/or E [29]. Ultrasound is suggested in DILI to exclude any focal changes to the liver and biliary obstruction [29], while liver biopsy determines parenchymal liver disease [29].	The incidence of DILI in Russia is 1–19 cases per million people per year. Nearly 10% of liver pathology-associated hospitalisations are drug induced [9].

ALD: Alcoholic liver disease; ALP: Alkaline phosphatase; ALT: Alanine aminotransferase; AST: Aspartate aminotransferase; CDT: Carbon-deficient transferrin; CLD: Chronic liver disease; CT: Computed tomography; DILI: Drug induced liver injury; DM: Diabetes mellitus; FIB-4: Fibrosis-4 index; FLI: Fatty liver index; GGTP: γ-Glutamyl transpeptidase; HCC: Hepatocellular carcinoma; hs-CRP: High sensitivity-C-reactive protein; MAFLD: Metabolic-associated fatty liver disease; MRI: Magnetic resonance imaging; NAFL: Non-alcoholic fatty liver; NAFLD: Non-alcoholic fatty liver disease; NASH: Non-alcoholic steatohepatitis; NFS: NAFLD liver fat score; RUCAM: Roussel-Uclaf Causality Assessment Method; TE: Transient elastography; WHO: World Health Organisation.

## Role of OS in CLD

Of note, OS acts as a common pathological factor in different phenotypes of CLD, leading to hepatic injury and progressive aggravation of the liver disease. Various underlying mechanisms contribute to OS in CLD, including mechanistic models that highlight cellular and molecular disease-related triggers or hits. The first hit due to insulin resistance (IR) and excessive free fatty acid (FFA) in circulation is followed by the second hit, including OS, lipid peroxidation, and mitochondrial dysregulation [20]. In the multiple parallel hit model, along with the earlier mentioned triggers, excess fatty acid-derived lipotoxic species are key to the substrate overload lipotoxic liver injury model that relies on OS and cellular pathology for disease progression [16,34]. Free fatty acids are generated by lipolysis of triglycerides or de novo by hepatocytes. They are also metabolized by β-oxidation pathways in the mitochondria or peroxisomes and converted into triglycerides. An imbalance in the production and metabolism of triglycerides leads to the accumulation of FFAs in the liver, causing endoplasmic stress, OS, the activation of inflammatory mediators. This can further lead to cell damage, cellular inflammation, hepatic stellate cell activation, and progressive fibrosis [35]. In addition, increased OS may also be associated with increased levels of serum lipopolysaccharides (LPS) due to intestinal bacterial translocation in patients with NAFLD [36,37]. Gut-derived endotoxins, in the case of ALD, initiate inflammatory responses and OS in liver tissue, both influencing independently and contributing to steatosis, steatohepatitis, and, ultimately, fibrosis [38,39]. The ROS and reactive nitrogen species (RNS) generated during alcohol consumption alter structurally and functionally the biological processes involved in liver injury. Consequently, hepatocytes are sensitized to the activity of cytokines such as tumour necrosis factor-α (TNF-α) and endotoxins, thereby activating signalling pathways [40]. Lipotoxicity due to excessive FFA, inflammation, and OS is a driving force for NAFLD [16,41]. Furthermore, increased lipid peroxidation and induced cytochrome P450 2E1 and serine kinases (c-Jun N-terminal kinases: JNK, inhibitor of nuclear factor kappa-B kinase subunit: IKK) fuel the progression of NAFLD. [42]. Excessive intrahepatic fat accumulation resulting from alterations in fat metabolism is often linked to OS, inflammation, and the generation of abnormal adipokines, which activates hepatocyte stress pathways and hampers signalling pathways [43,44]. The OS inherent in metabolic dysregulation derails several metabolic pathways such as IR [43] in the liver, leading to cell death, inflammation, and liver fibrogenesis [45]. The activation of metabolic or stress response pathways, including nuclear factor kappa-B (NF-КB), phosphatase and tensin homolog (PTEN), and microRNAs, often leads to HCC [42,46]. Additionally, fat accumulation owing to inflammation and comorbid conditions such as diabetes causes IR and hepatic necroinflammation by activation of Kupffer cells, leading to hepatic stellate cell activation, in turn, causing disease progression. Poor diet and genetic factors, including patatin-like phospholipase domain-containing protein 3 (PNPLA3) polymorphisms, enhance fat agglomeration in the liver, thus increasing the risk of fibrosis [42]. With respect to DILI, drugs and their reactive metabolites lead to a cascade of cellular events, including covalent bonds with mitochondria, leading to direct hepatic toxicity due to the accumulation of ROS/RNS, endoplasmic reticulum stress, and mitochondrial dysfunction. This subsequently leads to cell death [32,47,48]. Lipid peroxidation also adds to the stress mechanisms by impairing antioxidant defense mechanisms [47]. The role of mitogen-activated protein kinase has been established in mitochondrial stress, triggering mitochondrial membrane permeability transition and increasing the production of ROS. This ultimately causes DNA damage and cell death [47,49]. However, the extent of reactive metabolite formation and cellular stress varies based on the drug’s intrinsic properties and the host’s metabolism capacity, along with active defense mechanisms. Nuclear factor erythroid 2-related factor 2 (Nrf2) acts as a major defense mechanism in DILI [48,50].

Based on the available literature, it appears that hepatoprotection with an antioxidant reduces the liver damage caused by exogenous and endogenous hepatotoxic agents. Russian guidelines have recommended antioxidants for improving NAFLD, ALD, and DILI [5,8,9]. However, silymarin has been recommended for NAFLD and ALD, but for DILI, no such recommendation has been given [9].

## Guidelines for management of CLD in Russia

An effective treatment regimen should not only focus on reducing steatosis or CLD but also target underlying mechanisms and improve the metabolic dysregulation or risk factors linked with CLD [11]. Several international guidelines from Europe, the United States, and Russia, such as those from the EASL, the Russian Gastroenterological Association, and the Research Society of Gastroenterologists of Russia (NOGR), suggest lifestyle interventions such as increased physical activity, diet, and weight control for the management of CLD, along with pharmacological agents for symptomatic relief. This approach helps in restoring hepatic function, resolution of steatosis, and improves the quality of life of patients [5,11,27]. Bariatric and endoscopic metabolic therapies are also recommended in patients with MAFLD [11] in the absence of liver cirrhosis. Certain antidiabetic drugs, insulin sensitizers, statins, GLP-1a, phosphodiesterase inhibitors, and antioxidants (vitamin E) are suggested in patients with NAFLD and MAFLD to manage comorbid conditions [11,27]. However, pharmacotherapy should be reserved for patients with NASH and significant fibrosis [26]. The correction of nutritional status along with medicinal treatment has been recommended in the Russian guidelines for ALD [8]. Additionally, supportive antioxidant therapy has been proposed alongside drugs for correcting metabolic dysregulation or aetiological factors [13]. In particular, the use of silymarin for NAFLD and ALD has been recommended in Russian guidelines [5,8]. Table 2 elaborates on the comparison between Russian guidelines and other regional guidelines.

**Table 2.** Comparison of Russian Guidelines with other regional guidelines.

CLD phenotype	AASLD guidelines	EASL guidelines	APASL guidelines	Russian guidelines
ALD	The AUDIT and CAGE questionnaires are used along with alcohol biomarkers to aid in diagnosis. Relapse prevention medicines (naltrexone, acamprosate); cognitive-behavior therapy; and psychosocial therapy is recommended. Other treatment modalities are used in different forms of ALD, like alcoholic hepatitis, alcohol-associated steatosis. Liver transplant is undertaken is patients with decompensated alcohol-associated cirrhosis [10].	AUDIT questionnaire, MCV, AST/ALT, body mass index, LFT, non-invasive serum fibrosis test, and liver biopsy were used for diagnosis. Treatment modalities of different alcohol-associated liver disorder (alcoholic hepatitis, alcohol-associated fibrosis, alcoholic steatosis, alcoholic cirrhosis) have been elaborated in detail [12].	Various questionnaires are used to determine alcohol dependency, such as CAGE, MAST, and AUDIT. ALT, AST, AST/ALT, physical examination, hepatic imaging, and liver biopsy are used to establish the ALD diagnosis. Alcohol abstinence, naltrexone, or acamprosate in those who achieve abstinence, and nutritional therapy are recommended in ALD, while a separate treatment algorithm is followed for alcoholic hepatitis. Liver transplant in used in cirrhosis [89].	Ultrasound, TE, non-invasive fibrotests, direct laboratory markers (PEth, EtG, EtS, FAEE), and indirect markers (MCV, AST, AST/ALT, GGTP etc.) are used for diagnosis. Medicinal treatment for different ALD forms (alcoholic hepatitis), liver transplant, and rehabilitation is recommended. Additionally, silymarin is advised for use in ALD owing to its property to suppress lipid peroxidation [8].
NAFLD	Non-invasive assessment (NFS, FIB-4, EFL, serum biomarkers), imaging (vibration-controlled TE), and liver biopsy are used for diagnosis. Lifestyle intervention, insulin sensitisers, thiazolidinediones, vitamin E, bariatric surgery, and liver transplant are recommended [27].	Liver biopsy, ultrasound, biomarkers, scores of fibrosis, and HOMA-IR are used to diagnose NAFLD and associated metabolic syndrome. Diet and lifestyle modification, insulin sensitisers, antioxidants, cytoprotective, lipid-lowering agents, bariatric surgery, and liver transplant are recommended based on patient condition [26].	–	Imaging techniques; non-invasive diagnostic tests (Fibrotest, NAFLD fibrosis score, TE); and diagnostic liver biopsy are used as diagnostic aids. Metformin, pioglitazone, lipid-lowering agents, vitamin E, phospholipids, and UDCA along with other agents can be used as pharmacological treatment modalities. Silymarin use is also recommended [5].
DILI	Causality assessment and liver biopsy are conducted for diagnostic purposes. Discontinuation of the implicated agent, short-term administration of a bile acid resin, carnitine, N-acetylcystein, corticosteroids, along with other specific therapies according to causative agents, are used as treatment modalities [90].	Antibodies and HLA type tests are used for diagnosis of DILI and distinguish DILI and AIH. Injury from different drug categories had a different diagnostic approach. Liver biochemistry, imaging, and biopsy are recommended. Discontinuation of suspected agent and different targeted therapy for different agents are used (cholestyramine, carnitine, N-acetylcystein, UDCA, liver transplant) [29].	Laboratory testing, imaging, and biopsy are used for diagnosis. CDS and RUCAM scores are also employed. Withdrawal of suspected agent along with specific therapies (steroids, cholestyramine, carnitine, UDCA) is used as a management approach [91].	RUCAM scale is recommended for assessing risk of medication in DILI. Physical examination, laboratory and instrumental diagnostic techniques are used. Suspension of the causative agent is the primary step in the management of DILI. Medicinal agents (N-acetylcystein, L-carnitine, glycyrrhizic acid, L-ornithine L-aspartate, UDCA, ademetionine, glucocorticoids, phospholids) are used on a case-to-case basis [9].

ALD: Alcoholic liver disease; ALT: Alanine aminotransferase; AST: Aspartate aminotransferase; AUDIT: Alcohol use disorders identification test; CDS: Clinical Diagnostic Scale; DILI: Drug induced liver injury; EFL: Enhanced Liver Fibrosis; EtG: Ethyl glucuronide; EtS: Ethyl sulphate; FAEE: Fatty acid ethyl esters; FIB-4: Fibrosis-4 index; GGTP: γ-Glutamyl transpeptidase; HLA: human leukocyte antigen; HOMA-IR: Homeostatic Model Assessment of Insulin Resistance; LFT: Liver function test; MAFLD: Metabolic-associated fatty liver disease; MAST: Michigan Alcohol Screening Test; MCV: mean corpuscular volume; NAFLD: Non-alcoholic fatty liver disease; NFS: NAFLD liver fat score; PEth: Phosphatidylethanol; RUCAM: Roussel-Uclaf Causality Assessment Method; TE: Transient elastography; UDCA: Ursodeoxycholic acid.

## Silymarin as a pharmaceutical agent

The panel critically evaluated the antioxidant properties of the original silymarin as a hepatoprotective agent and laid the foundation for using original silymarin for treating CLD. Silymarin, derived from the dried seeds and fruits of the milk thistle plant, also known as *Silybum marianum* (*Silybi mariani fructus extractum*), has been used medicinally as an antioxidant and biological agent due to its chemical constituents (polyphenols, flavonolignans, and flavonoids) [21]. The most prevalent and biologically active flavonolignan, viz. silibinin (also called silybin), undergoes phases I and II hepatic biotransformation [21]. The crude silymarin extract is lipophilic and rapidly absorbed following oral administration [21]. The pharmacological properties of silymarin are based on its ability to target elevated liver enzymes, suppress OS, prevent the activation of hepatic stellate cells, and activate immune cells (Kupffer cells), thereby reducing oxidative damage, fibrosis, and inflammation [21]. The duration of therapy with silymarin can vary depending on the disease, severity, extent of clinical and laboratory abnormalities, and individual patient requirements. Silymarin acts as a scavenger of ROS, augments the level of glutathione in the liver, and inhibits hepatic NF-КB activation [21]. Silymarin suppresses lipid peroxidation, inhibits the formation of free radicals, and stimulates the synthesis of proteins and phospholipids within hepatocytes. These actions, in turn, protect against cellular damage, stabilize the cell membrane, and decrease membrane permeability [8]. The phenolic structure of silymarin enables it to form stable compounds with ROS, which forms the basis of its hepatoprotective and antioxidant properties [22]. Silymarin’s properties as an antifibrotic, anti-inflammatory agent and IR modulator have been studied in preclinical and *in vitro* models [51–54], thus establishing it as a hepatoprotective agent. Further, studies also suggest the potential of silymarin to accumulate in the liver and plasma following oral administration at physiological amounts [55]. Additionally, the hepatoprotective activity of silymarin has been reported at lower and higher doses than the recommended dose of 140 mg three times a day [5].

Therefore, based on pharmacodynamic, pharmacokinetic properties, and the quality of the original silymarin prepared, the expert panel reached an agreement on its untapped potential as a safe hepatoprotective agent [56].

### Clinical effectiveness of silymarin in liver diseases

The effectiveness of original silymarin at a maximum dose of 140 mg three times a day in the therapeutic regimen of CLD needs to be established based on clinical evidence and real-life studies. The panel undertook a discussion to confirm the clinical benefits of original silymarin. The meta-analysis conducted by de Avelar *et al.* wherein randomised and controlled clinical trials were included (six articles used), advocates the use of silymarin in reducing serum levels of ALT and AST [57]. The meta-analysis conducted by Saller *et al.* wherein 36 articles were used for the examination, also confirmed the use of silymarin in liver diseases, because of the improvement in liver function tests and reduction in mortality rates following the administration of silymarin [58]. The meta-analysis by Tao *et al.* wherein 585 patients from five randomised clinical trials were treated with silymarin for DILI, showed that silymarin exerted protective activity, measured by liver function tests, in patients undergoing anti-TB treatment. It also reduced the occurrence of anti-TB DILI four weeks post-silymarin initiation [59]. Similarly, a meta-analysis of eight published randomised clinical trials (using PRISMA) established the positive efficacy of silymarin in reducing transaminases levels in patients with NAFLD [60].

A systematic review of 5 clinical trials involving 602 patients with ALD and liver cirrhosis showed a 57.8% reduction in liver-related mortality in patients taking silymarin compared to placebo [58,61–64]. Even though silymarin does not affect the viral load, it may be considered useful as a supportive treatment alongside standard antiviral agents for improving liver enzyme levels [65].

It was reported in an observational study that original silymarin was effective in patients with elevated baseline liver enzymes due to prolonged use of potentially hepatotoxic drugs. A large proportion of patients who took original silymarin 420 mg/day for four months, experienced significant improvement in terms of liver enzymes, betterment of symptoms, and overall quality of life [66].

In a randomised controlled pilot study, silymarin was used along with other drugs to improve biochemical indices in patients with NAFLD [67]. A double-blind controlled trial has confirmed the effects of silymarin on chemical, functional, and morphological alterations in the liver.[68] A randomised, double-blind, placebo-controlled trial in patients with a biopsy-proven NASH and NAFLD activity score of 4 or more showed a significant reduction in the fibrosis score in patients receiving silymarin (700 mg per day) for 48 weeks. Silymarin was reported to be safe, with even high doses being well tolerated [56]. Similarly, a higher dose of silymarin was used in patients with alcoholic cirrhosis in a double-blind randomised control [69] and for improving metabolic dysregulation in patients with cirrhotic diabetes, as observed in an open, controlled trial [70]. Lower doses of silymarin (<140 mg three times a day) were also used clinically to establish its hepatoprotective and antioxidant activity, either alone or along with other agents, as evident in an open, controlled trial; randomised controlled trials; and pilot study [71–73]. Randomised clinical trials have also demonstrated the safety and effectiveness of silymarin in reducing the levels of liver enzymes [74] and in restoring superoxide dismutase [75] in patients with DILI. Furthermore, the effectiveness of silymarin in combination with other agents was established in patients with NAFLD in a randomised double-blinded, placebo-controlled trial [76] and randomised controlled trials [77–79], wherein improvement in liver enzymes, metabolic markers, oxidative stress, endothelial dysfunction, total cholesterol, and homeostasis model was evident, without leading to any serious specific side effects. Certain other clinical trials also demonstrated the use of silymarin in the management of CLD, either as monotherapy or in combination [61,63,64,80–83].

## Limitations of the present work

Furthermore, the present work is associated with certain shortcomings, including inadequate clinical evidence and limited data about the use of silymarin. Furthermore, the present work does not systematically categorize the level of available clinical evidence for the benefits of silymarin. Additionally, the opinions of the panel members may not necessarily represent a global viewpoint in this regard. Based on the available clinical evidence, the expert panel formulated expert statements regarding the benefits of antioxidant treatment with silymarin for CLD. There is, however, still comprehensive work to be done to understand the mechanisms and mediators of clinical benefits. Current knowledge gaps in this domain demands necessitate further research into silymarin's clinical efficacy for CLD.

## Panel’s perspective on the use of silymarin

To achieve an antifibrotic effect, silymarin should be used at a dose of 140 mg three times a day for 6–12 months. As per the suggestion of the panel, it is reasonable to use original silymarin as an anti-inflammatory agent along with ursodeoxycholic acid or ademetionine. Additionally, the panel suggested that in patients with inflammation and fibrosis of the liver, original silymarin must be taken for at least 12 months. Silymarin can be used in the complex treatment of patients with viral hepatitis, because as an antioxidant, it reduces toxic load, cytolysis syndrome, and improves the quality of life. Recent progress in treating DILI with original silymarin is very encouraging. For managing ALD, alcohol needs to be eliminated before contemplating hepatoprotection with silymarin. There is a clear clinical rationale for using an antioxidant such as silymarin as a hepatoprotective and hepatotropic agent. Further, silymarin treatment may begin early and may be prolonged with careful monitoring of liver enzymes. The expert panel opined that original silymarin would have a clear advantage over other marketed generic formulations in terms of its high concentration, bioavailability, safety, effectiveness, and quality. Further, a large body of scientific evidence built on original silymarin studies can be leveraged in this regard. The panel also opined that as original silymarin is a safe and effective drug and as there exists an evidence base on its clinical and biochemical effects, it can be recommended for the treatment of CLDs.

## Expert opinion statement

The expert panel reviewed existing evidence and agreed on the hepatoprotective and hepatotropic benefits conferred by 140 mg original silymarin three times a day, which acts by targeting OS in CLDs such as ALD, NAFLD/NASH, MAFLD, and DILI. The treatment duration can be from 2–3 months for mild cases to at least 12 months for severe cases.

## Conclusion

The protection of the liver from long-term toxic damage is a prerequisite for overcoming any kind of hepatic injury. The key pathophysiological mechanism underlying hepatic damage in all clinical phenotypes of CLD (DILI, ALD, NAFLD, MAFLD, NASH, and liver cirrhosis) is OS; hence, treatment with antioxidants may have a significant role in hepatoprotection. Most of the biochemical and clinical evidence evaluated by the expert panel used original silymarin, which was shown to be safe and effective at a dose of 140 mg three times a day. Clinically, there is a need for greater evidence on the long-term use of silymarin for concomitant diseases (such as NAFLD and ALD), in the context of CLDs. For NAFLD, silymarin should be given at the maximum dose (140 mg, three times a day), as indicated by the manufacturer. Generic manufacturing may lead to deviations from the original silymarin formulation in terms of composition, bioavailability, safety, and effectiveness. Therefore, for reproducible clinical outcomes, it is advisable to use original silymarin or Legalon® as a hepatoprotective and hepatotropic agent for prophylaxis and management of CLDs.

## Figures and Tables

**Table:** 

Author Name	Responsibilities						
	Criteria 1			Criteria 2		Criteria 3	Criteria 4
	Conception and design	Acquisition of data	Analysis and interpretation of data	Drafting of the manuscript	Critical revision of the manuscript for important intellectual content	Final approval	Agreement to be accountable for all aspects of the work
Aghemo Alessio	Yes	Yes	Yes	Yes	Yes	Yes	Yes
Alekseeva Olga P			Yes		Yes	Yes	Yes
Angelico Francesco	Yes	Yes	Yes	Yes	Yes	Yes	Yes
Bakulin Igor G	Yes	Yes	Yes	Yes	Yes	Yes	Yes
Bakulina Natalia V			Yes		Yes	Yes	Yes
Dmitry Bordin			Yes		Yes	Yes	Yes
Bueverov Alexey O			Yes		Yes	Yes	Yes
Drapkina Oxana M			Yes		Yes	Yes	Yes
Gillessen Anton	Yes	Yes	Yes	Yes	Yes	Yes	Yes
Kagarmanova Elvira M			Yes		Yes	Yes	Yes
Korochanskaya Natalia V			Yes		Yes	Yes	Yes
Kucheryvii UA			Yes		Yes	Yes	Yes
Lazebnik Leonid S	Yes	Yes	Yes	Yes	Yes	Yes	Yes
Livzan Maria A			Yes		Yes	Yes	Yes
Maev Igor V			Yes		Yes	Yes	Yes
Martynov Anatolii I	Yes	Yes	Yes		Yes	Yes	Yes
Osipenko Marina F			Yes		Yes	Yes	Yes
Sas Evgenii I			Yes		Yes	Yes	Yes
Starodubova Antonina			Yes		Yes	Yes	Yes
Uspensky Yurii P			Yes		Yes	Yes	Yes
Vinnitskaya Elena V	Yes	Yes	Yes		Yes	Yes	Yes
Yakovenko Emilia P			Yes		Yes	Yes	Yes
Yakovlev Alexey A			Yes		Yes	Yes	Yes

## Data Availability

Data sharing is not applicable to this article, as no new data were created or analysed in this study.
